# Alexithymia as a Transdiagnostic Precursor to Empathy Abnormalities: The Functional Role of the Insula

**DOI:** 10.3389/fpsyg.2017.02234

**Published:** 2017-12-21

**Authors:** Andrew Valdespino, Ligia Antezana, Merage Ghane, John A. Richey

**Affiliations:** Social Clinical Affective Neuroscience Laboratory, Department of Psychology, Virginia Tech, Blacksburg, VA, United States

**Keywords:** alexithymia, insula, empathy, psychiatric disorders, affective neuroscience

## Abstract

Distorted empathic processing has been observed across multiple psychiatric disorders. Simulation theory provides a theoretical framework that proposes a mechanism through which empathy difficulties may arise. Specifically, introspection-centric simulation theory (IST) predicts that an inability to accurately interpret and describe internal affective states may lead to empathy difficulties. The purpose of this review is to synthesize and summarize an empirical literature suggesting that simulation theory provides insights into a cognitive and neurobiological mechanism (i.e., alexithymia and insula pathology) that negatively impacts empathic processing, in addition to how disruptions in these processes manifest across psychiatric disorders. Specifically, we review an emerging non-clinical literature suggesting that consistent with IST, alexithymia and associated insula pathology leads to empathy deficits. Subsequently, we highlight clinical research suggesting that a large number of disorders characterized by empathy pathology also feature alexithymia. Collectively, these findings motivate the importance for future work to establish the role of alexithymia in contributing to empathy deficits across clinical symptoms and disorders. The current review suggests that simulation theory provides a tractable conceptual platform for identifying a potential common cognitive and neural marker that is associated with empathy deficits across a wide array of diagnostic classes.

## Introduction

Behavioral and neural empathy deficits characterize many psychiatric conditions, including autism spectrum disorder (ASD; Minio-Paluello et al., 2009), psychopathy (Decety et al., 2013), borderline personality disorder (BPD) (Dziobek et al., 2011), and narcissistic personality disorder (NPD) (Ritter et al., 2011). This suggests that empathy is a relevant transdiagnostic dimension with implications for functional impairment and treatment development. Empathy refers to sharing an isomorphic affective state to others, to understand their feelings (de Vignemont and Singer, 2006). Introspection-centric simulation theory (IST) predicts that empathy requires introspecting upon an isomorphic internal state (Shanton and Goldman, 2010). Empirical work on alexithymia provides a platform for evaluating whether introspection deficits link to empathy difficulties in clinical and typical populations. Alexithymia describes an inability to articulate and interpret internal feelings (Sifneos, 1973). Since this original conceptualization, multiple alexithymia subtypes have been proposed (Bermond et al., 2007), which has led to a distinction between cognitive (i.e., difficulties verbalizing and identify emotions) and affective (low awareness of emotional arousal) alexithymia.

An IST framework predicts that alexithymia may lead to empathy deficits. That is, while alexithymia and empathy differ in that they pertain to internally versus externally oriented states, respectively, they share common referents (i.e., feelings and emotions). Refer to Bird and Viding (2014) for a theoretical review that proposes the role of simulation in empathy, in the context of autism, psychopathy, and alexithymia. The growing alexithymia literature allows for the evaluation of its effects on empathy at behavioral and neural levels. Therefore, the purpose of this review is to summarize theoretical and empirical work that suggests (1) consistent with IST, alexithymia contributes to empathy deficits (2) the insula is a biomarker of alexithymia deficits, and (3) alexithymia presents as a candidate contributor to empathy deficits across multiple psychiatric disorders.

**FIGURE 1:**
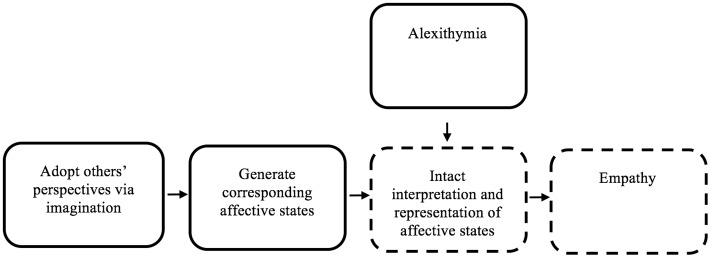
The role of alexithymia in empathy deficits, in the context of introspection-centric simulation theory. This figure represents a schematic simplified version of Goldman’s simulation theory (Goldman, 1992). Alexithymia is added in the figure, to clarify how alexithymia might yield empathy deficits. Imagination is used to simulate the perspective of an individual. Then, affective states are generated that follow from the simulated state. Those affective states are then represented in the ‘simulator,’ yielding empathy. Alexithymia may cause a disruption in the accurate interpretation and representation of affective states, causing empathy deficits. Dotted-lines indicate nodes within an IST framework that may plausibly be negatively impacted by alexithymia.

Simulation theory posits that simulation is leveraged as a predictive device, such that a social agent simulates what they might do in a comparable situation, or simulates a matching internal state, then utilizes that information to adopt another’s perspective (see **Figure 1**; Goldman, 1992). Multiple forms of simulation theory have been proposed (see Shanton and Goldman, 2010 for a discussion of alternative simulation theory perspectives). IST posits that generating and introspecting upon a matching internal state is required for empathy (Goldman, 2006). Therefore an IST account suggests that since alexithymia involves a distorted representation of internal affective states, empathy deficits should co-occur.

## Behavioral Findings Linking Alexithymia with Decreased Empathy

Consistent with IST, behavioral findings suggest that alexithymia is correlated with and leads to empathic deficits. Silani et al. (2008) found correlations between alexithymia and empathy across both typical and clinical populations (see Supplementary Table 1 for a summary of research findings included in this review). Links between alexithymia and empathy have also been found in the context of empathizing based on facial expressions. Specifically, in a study by Moriguchi et al. (2006), participants were asked to rate the experienced pain of individuals based on their facial expression. High alexithymia participants ascribed less experienced pain, suggesting that difficulties perceiving one’s internal affective state may blunt the perception others’ affective states. Alexithymia also mediated the relationship between one’s ability to describe and act with mindful awareness and empathy (MacDonald and Price, 2017).

## The Role of the Insula in Alexithymia and Concurrent Empathic Deficits

Alexithymia is a complex neurofunctional phenomenon arising from multiple brain regions (Moriguchi and Komaki, 2013). It is not the purpose here to claim that the insula is the only region underlying alexithymia. It is out of the scope of this review to evaluate the functional role of all regions within the context of IST, alexithymia, empathy, and psychiatric disorders. Given the large body of research suggesting the insula plays a functional role in inner affective experience awareness [Lang, 1994; Craig, 2009; Zaki et al., 2012; Damasio and Carvalho, 2013; Bird and Viding, 2014), we focus our review on the insula.

Empirical work suggests that insula pathology is associated with alexithymia. Specifically, increased left insula glutamate concentrations positively correlated with alexithymia (Ernst et al., 2013). Increased insula dopaminergic receptor availability also positively correlated with alexithymia (Okita et al., 2016). Additionally, alexithymia is associated with decreased gray matter insula volume (Goerlich-Dobre et al., 2014; Laricchiuta et al., 2014). Finally, voxel-based morphometry studies suggest that insula pathology may lead to a domain-specific deficit in cognitive (not affective) alexithymia (Goerlich-Dobre et al., 2014, 2015).

To date, one study assessed for a causal link between insula pathology and alexithymia. Hogeveen et al. (2016) measured alexithymia severity across three groups characterized by increasing insula damage. Insula damage severity predicted increased alexithymia.

Within an IST account, given that accurate processing of internal affective states is requisite for empathy, and given that the insula is implicated in alexithymia, it would be expected that insula pathology should cause empathic deficits. One neuroimaging study assessed the modulating role of alexithymia on insula responsiveness and empathy (Bird et al., 2010). In this fMRI study, participants empathized with a partner who experienced physical pain. Left anterior insula activity was positively associated with level of empathy. Furthermore, the association between left anterior insula response and empathy was weaker in high relative to low alexithymia participants.

Overall, empirical work spanning structural, neurochemical, and functional neuroimaging modalities suggests that insula pathology may lead to alexithymia and associated empathy deficits. Furthermore, very preliminary work provides clues that insula pathology may be associated more with difficulties identifying, analyzing, and verbalizing feelings (i.e., cognitive alexithymia). Replication across structural and functional neuroimaging modalities are needed to support the possible functional specificity of the insula in the context of alexithymia.

## The Role of the Insula in Alexithymia and Empathy Deficits Across Psychiatric Disorders

The literature reviewed thus far suggests that alexithymia leads to empathy deficits in typical populations. This suggests that IST provides a framework for assessing whether alexithymia leads to empathy deficits in clinical populations. To date, while little empirical work has assessed whether empathy deficits source from alexithymia across disorders, we review a body of work suggesting that a variety of psychiatric presentations featuring empathy deficits, exhibit co-occurring alexithymia. This literature highlights the importance for future work to establish the mechanistic role of alexithymia in contributing to empathy deficits across clinical presentations.

### Autism Spectrum Disorder

Alexithymia prevalence amongst ASD is higher than in the general population (Hill et al., 2004). ASD may also be characterized by greater deficits in cognitive, relative to affective forms of alexithymia (Berthoz and Hill, 2005; Silani et al., 2008). This is interesting given an emerging body of research suggesting that ASD is also characterized by greater struggles with cognitive empathy (i.e., comprehending and adopting the emotional perspective of others), relative to affective empathy (i.e., actually feeling others’ emotional state; Deschamps et al., 2014).

Empathy deficits have also been observed in ASD (Baron-Cohen and Wheelwright, 2004; Sucksmith et al., 2013). Regarding a link between alexithymia and empathy deficits in ASD, Bird et al. (2010) operationalized empathy as insula activation magnitude while observing others experiencing pain. Insula activation negatively correlated with alexithymia during the empathy condition. After controlling for alexithymia severity in ASD and controls, no group difference in empathy was evident. The authors concluded that alexithymia, rather than ASD severity, drives empathic deficits. Given that alexithymia estimates are disproportionately higher in ASD (Hill et al., 2004), this suggests that alexithymia may be an important mechanism accounting for empathy deficits in this population.

Silani et al. (2008) also found that the insula was implicated in alexithymia and diminished empathy in ASD. Specifically, for both groups, during affective introspection, a positive correlation between bilateral insula parameter estimates and empathy, and a negative correlation with alexithymia were observed. In addition, at a more relaxed statistical threshold, ASD adults demonstrated hypoactivation of the anterior insula while rating how pleasant or unpleasant they felt after viewing negative affective images.

Overall, these findings suggest that alexithymia and related insula processing in ASD may be associated with decreased empathy. Furthermore, results suggest that the subgroup of ASD individuals high in alexithymia, may be specifically characterized by the cognitive subtype. Preliminary results should therefore not be interpreted as attenuated arousal to emotional events, but rather a specific difficulty with identifying and verbalizing feelings.

### Psychopathy

Psychopathy refers to personality traits characterized by callousness, lack of concern about performance, and diminished affect (Blair, 2013). Given that diminished empathy is a core feature of psychopathy (Hare, 1980), IST provides an inroads to assess whether empathic deficits partly source from alexithymia. Psychopathy has been associated with alexithymia (Kroner and Forth, 1995; Louth et al., 1998). Regarding a link between alexithymia and empathy, results are inconsistent with respect to IST. Specifically, alexithymia has been associated with ‘secondary’ psychopathy, which is not typically characterized by empathy deficits (i.e., emotional instability, risk-taking behavior, and impulsivity) and less with ‘primary’ psychopathy (i.e., empathy deficits, manipulativeness, fear insensitivity, callousness; Grieve and Mahar, 2010; Lander et al., 2012). However, studies show that both primary and secondary psychopathy are associated with reduced affective empathy (Wai and Tiliopoulos, 2012). Currently, no studies directly address the cognitive/affective alexithymia and cognitive/affective empathy relationship, making it difficult to draw structured conclusions.

Consistent with IST, Jonason and Krause (2013) found that increased psychopathic traits were associated with increased alexithymia, and decreased empathy. Specifically, while deficits existed across multiple alexithymia subdomains, only externally oriented thinking (i.e., preference to attend to external information rather than internal states) was related to affective empathy (i.e., feeling others’ emotional states). It is unknown whether alexithymia mediates the relationship between psychopathy and empathy, however, alexithymia mediated the relationship between criminal aggression and empathy (Winter et al., 2017).

No psychopathy studies have assessed whether insula pathology causes alexithymia. However, research suggests that the insula plays a role in empathic reasoning in psychopathic populations. Specifically, when imagining others in pain, high psychopathy predicted diminished insula activity (Decety et al., 2013; Marsh et al., 2013). High psychopathy individuals also showed attenuated insula response while identifying a perpetrator’s emotional state (Decety et al., 2015), and when observing affection or exclusion toward others (Meffert et al., 2013). Insula response to empathy for pain may also be differentrially dependent on facets of psychoapthy. Specifically, affective-interpersonal psychopathy traits were negatively associated with insula response while empathizing, where as lifestyle-antisocial trait were positively associated (Seara-Cardoso et al., 2015).

Results suggest a complex presentation of alexithymia and empathy in psychopathy. Specifically, in secondary psychopathy, characterized by disinhibition, cognitive alexithymia is associated with affective empathy deficits. While there is evidence of affective empathy deficits in primary psychopathy, cognitive alexithymia has not been found, suggesting deficits source either from an alexithymia-independent mechanism or possibly a relationship with deficits in affective alexithymia. Furthermore, while there is evidence of attenuated insula response during cognitive and affective empathy tasks, this atypicality seems specific to the social role of the other. Additionally, given the possible causal role of the insula in alexithymia development (Hogeveen et al., 2016), future work in psychopathy should determine whether affective or cognitive empathy deficits and insula pathology are differentially mediated by cognitive or affective alexithymia.

### Narcissistic Personality Disorder

Narcissistic personality disorder is characterized by grandiosity, self-admiration needs, and empathy deficits (American Psychiatric Association, 2013). An emerging body of work suggests that narcissism is particularly associated with an alexithymia subtype (i.e., difficulties identifying feelings). Specifically, Schimmenti et al. (2017) found that high levels of narcissistic traits were associated with difficulties identifying feelings, and not with other alexithymia subtypes. Jonason and Krause (2013) found that across the Dark Triad Traits (i.e., narcissism Machiavellianism, psychopathy), narcissism uniquely predicted difficulties identifying feelings. There is also evidence that specific facets of narcissism may be associated with distinct alexithymia subtypes. Specifically, narcissistic defenses (i.e., seeing others as wrong and abusive) correlated with difficulties identifying feelings, whereas core narcissism (i.e., grandiosity and entitlement) was linked to difficulties describing feelings to others. The majority of studies in this area do not leverage clinical NPD samples, but rather assess narcissistic traits in typical samples. However, preliminary work in the form qualitative research does suggest that difficulties identifying feelings extend to clinical NPD samples also (Dimaggio et al., 2007).

While results are mixed, there is growing evidence that NPD may be characterized by emotional empathy deficits (i.e., feeling an emotional response to another person’s state), with cognitive empathy being relatively intact (Baskin-Sommers et al., 2014). It should be noted though that cognitive empathy deficits have been found, particularly for the emotions fear and disgust (Marissen et al., 2012). It has become increasingly appreciated that given the tendency of narcissistic individuals to over-estimate their empathic ability, objective measures of empathy that bypass self-report may provide a more valid measure of empathy in this population (Ritter et al., 2011). This suggestion is consistent with the lack of empathy deficits that have been found on measures of self-report (Fan et al., 2011; Marissen et al., 2012) contrasted with pronounced deficits that have been documented using performance-based empathy measures (Ritter et al., 2011).

With regards to insula functioning, NPD evidenced decreased insula gray matter volume, and further, gray matter volume in the insula negatively correlated with empathic ability (Schulze et al., 2013). Fan et al. (2011) found that narcissism symptomatology was associated with increased levels of alexithymia, as well as reduced positive signal change from non-empathy to empathy task conditions in the right anterior insula.

Overall, while no research has yet assessed whether alexithymia leads to empathy deficits in narcissistic individuals, the co-occurrence of these difficulties indicates this may be a promising avenue of research. The particularly pronounced difficulties with describing internal feelings, suggests that this particular alexithymia subtype may be a useful first step in establishing whether a causal role of between alexithymia in empathic difficulties exists.

### Borderline Personality Disorder

Borderline personality disorder is characterized by affective impulsivity and instability relating to social relationships and the self (American Psychiatric Association, 2013). Affective dysregulation is a prominent BPD feature (Glenn and Klonsky, 2009). Interestingly, dysregulation may also be mechanistically related to empathic deficits. Specifically, increased arousal has been observed in BPD while empathizing, and further, decreased emotional dysregulation has been associated with diminished empathetic concern (Dziobek et al., 2011; Kalpakci et al., 2016).

With respect to IST, it is possible that emotional dysregulation exacerbates alexithymia, causing empathic deficits. Consistent with this possibility, emotional dysregulation and alexithymia are conceptually and mechanistically related. Specifically, each pertains to the distorted processing of internal affective experience. Furthermore, increased aversive inner tension (which is related to emotional dysregulation) has been associated with difficulties identifying internal emotions in BPD (Wolff et al., 2007). These findings are consistent with Ridings and Lutz-Zois (2014), who found that alexithymia correlated with both emotional dysregulation, and BPD tendencies. Regarding a mechanistic link between alexithymia and empathy, Flasbeck et al. (2017) found that the effect of early life adversity on empathy for psychological pain was mediated by alexithymia in BPD. Overall, while significant work would be required to scrutinize this speculative IST prediction of the link between affective dysregulation, alexithymia, and diminished empathy, the purpose here is to highlight the utility of IST, and the potential role of alexithymia in BPD empathic deficits.

While no studies currently exist on the neural correlates of alexithymia in BPD, neuroimaging findings suggest that aberrant insula processing may contribute to dysregulated negative affect in BPD, with implications for altered empathic processing. Specifically, BPD has been associated with diminished insula activity while viewing negative social–emotional stimuli (Koenigsberg et al., 2009; Frick et al., 2012), and increased activity to physical pain after social rejection (Bungert et al., 2015). Regarding empathic processing, BPD evidenced increased insula activity relative to controls during emotional (but not cognitive) empathy (Dziobek et al., 2011). Given that no neuroimaging studies currently exist on alexithymia in BPD, further work may assess whether insula dysregulation in BDP might reflect inaccurate or dysregulated appraisals of internal feelings. Consistent with this possibility, preliminary related work suggests that insula pathology in BPD may confer difficulties generating internally and externally relevant social information. Specifically, the insula mediated poorer consistency in rating the self and others in BPD (Beeney et al., 2016). Furthermore, dissociative tendencies in BPD (which reflect detachment from internal experience) were associated with altered functional connectivity in the insula (Wolf et al., 2011).

## Clinical Implications, Limitations, and Directions for Future Research

Consistent with IST, research supports a link between alexithymia and associated insula pathology, and empathy deficits in typical populations. Furthermore, multiple clinical presentations exhibit co-occurring alexithymia and empathy deficits. Combined with reviewed preliminary mediation findings in some clinical presentations, these results indicate that alexithymia is a potential transdiagnostic source of empathic difficulties.

There are limitations to this review worth mentioning. Given that a review focus was the insula, many brain regions that have been linked with alexithymia were not discussed (Moriguchi and Komaki, 2013). An additional limitation stems from the small number of mediation studies to comprehensively evaluate IST predictions in psychiatric disorders.

In the future, levering varied alexithymia measurement approaches, in addition to novel measurement development, may elucidate the role of alexithymia subtypes across disorders. The majority of studies reviewed leveraged the Toronto Alexithymia Scale (Taylor et al., 1992) as their alexithymia operationalization. This measure provides nuanced information about cognitive alexithymia, but unlike measures such as the Bermond–Vorst Alexithymia Questionnaire (Vorst and Bermond, 2001), it does not address the cognitive and affective alexithymia distinction.

A central goal of this review was to provide an empirical basis for the significant potential utility in continued research clarifying the transdiagnostic link between alexithymia and empathy. Currently, alexithymia does not form a core treatment component for the disorders reviewed. Alexithymia is a well-specified, empirically measurable construct. Therefore, alexithymia is suited for empirical clinical work into therapeutic mechanisms of change, as well as in clinical practice settings for purposes of treatment outcome monitoring.

## Author Contributions

AV conceptualized the review. LA, MG, and JR provided significant conceptual refinement. AV, LA, and MG drafted the manuscript. LA, MG, and JR provided critical revisions. All authors approved the final manuscript.

## Conflict of Interest Statement

The authors declare that the research was conducted in the absence of any commercial or financial relationships that could be construed as a potential conflict of interest.
