# Supplementary material for: Alexithymia as a Transdiagnostic Precursor to Empathy Abnormalities: The Functional Role of the Insula
Source: Front Psychol. 2017 Dec 21;8:2234. doi: 10.3389/fpsyg.2017.02234 (PMC5742870; doi:10.3389/fpsyg.2017.02234)
Supplement: Supplementary file 1 [file Table_1.pdf]

Supplementary Material

Alexithymia as a transdiagnostic precursor to empathy abnormalities: the functional role of the insula

Andrew Valdespino*[1], Ligia Antezana[1], Merage Ghane[1], & John A. Richey[1]

[1]Social Clinical Affective Neuroscience Laboratory, Department of Psychology, Virginia Tech, Blacksburg, VA, USA

*** Correspondence:**
Andrew Valdespino
andrewdv@vt.edu

1 Supplementary Table

Table 1. Summary of research

Article	Number of participants	Tools	Results
Behavioral findings linking alexithymia with decreased empathy			
(MacDonald & Price, 2017)	Undergraduate students (n=616)	Self-report measures (Toronto Alexithymia Scale – TAS-20, Five-Facet Mindfulness Questionnaire, Questionnaire of Cognitive & Affective Empathy)	Alexithymia mediated the relationship between describing and acting with awareness (mindfulness) and cognitive empathy.
(Moriguchi et al., 2006)	Alexithymia group (n=16), non-	Self-report measures (Toronto Alexithymia Scale – TAS-20,	Individuals with alexithymia had lower

	alexithymia group (n=14)	Interpersonal Reactivity Index – IRI, Emotional Empathy Scale – EES), structured-interview (modified Beth Israel Hospital Psychosomatic Questionnaire for alexithymia – SIBIQ)	pain ratings and empathy scores.
(Silani et al., 2008)	ASD (n=15), controls (n=15)	Self-report measures (Toronto Alexithymia Scale – TAS-20, Bermond-Vorst Alexithymia Questionnaire – BVAQ, Interpersonal Reactivity Index – IRI), fMRI	For ASD and controls, negative correlation between the TAS-20 and IRI.
The role of the insula in alexithymia and concurrent empathic deficits			
(Bermond et al., 2007)	Students of various nationalities and groups, including Dutch (n=375), English (n=175), Australian, (n=375), Belgian (n=175), Italian (n=791), Polish (n=427), Russian (n=141), and university employees and visitors (n=216)	Self-report measures (Bermond-Vorst Alexithymia Questionnaire – BVAQ)	Five subscales of the BVAQ resulted in a two-factor solution in all cultural groups and explained between 55-66% of the variance. These two factors (i.e., cognitive and affective alexithymia) were independent rather than correlated.
(Bird et al., 2010)	ASD and control participants combined and divided into low alexithymia (n=18) and high alexithymia (n=18) groups.	Self-report measures (Toronto Alexithymia Scale – TAS-20, Bermond-Vorst Alexithymia Questionnaire – BVAQ, Interpersonal Reactivity Index – IRI), empathy for pain task, fMRI	Empathic brain responses were associated with increased left anterior insula activation. The response magnitude predicted alexithymia severity in ASD and controls, and did not vary as a function of group.

(Craig, 2009)	Review paper	Review paper	Evidence suggesting role of the anterior insula in the re-representation of interoception and involvement in all subjective feelings. Argues for role of anterior insula in awareness and as a neural correlate of consciousness.
(Damasio & Carvalho, 2013)	Review paper	Review paper	Discusses the role of the insula in sensing and mapping body states and generating feelings. Also discusses the role of the insula in modulating the processing and experience of interoceptive and emotional states, connecting them with memory, language, and reasoning-related regions.
(Ernst et al., 2013)	Healthy adults (n=19)	Self-report measures (Toronto Alexithymia Scale – TAS-20), proton magnetic resonance spectroscopy	Glutamate levels in the left insula were positively associated with alexithymia.
(Goerlich-Dobre et al., 2014)	High cognitive alexithymia (n=20), low cognitive alexithymia (n=20)	Self-report measures (Bermond-Vorst Alexithymia Questionnaire – BVAQ, Toronto Alexithymia Scale – TAS-20), Voxel-based morphometry (MRI)	Larger grey matter volume in right posterior insula in high cognitive relative to low cognitive alexithymia individuals. Low emotional reactivity on affective alexithymia measure was associated with larger grey matter volume in right cingulate.

(Goerlich-Dobre, Votinov, Habel, Pripfl, & Lamm, 2015)	Adults subdivided into different groups based on type of alexithymia. Type I, high cognitive, high affective (n=30); Type II, high cognitive, low affective (n=32); Type III, low cognitive, high affective (n=22), Lexithymics, low cognitive, low affective (n=34)	Self-report measures (Bermond-Vorst Alexithymia Questionnaire – BVAQ), Voxel-based morphometry (MRI)	Those with cognitive and affective, or cognitive only alexithymia, showed reduced left amygdala and thalamus volumes. The cognitive alexithymia group only showed reduced volume in right amygdala, left posterior insula, precuneus, caudate, hippocampus, and parahippocampus. The affective alexithymia group only showed medial cingulate volume reduction, and larger anterior cingulate.
(Hogeveen, Bird, Chau, Krueger, & Grafman, 2016)	Non-brain injured controls (n=33), brain-injured participants, (n=129). Patients further separated into insula damage groups: 0% anterior insula damage (n=89), > 0% and < 15% anterior insula damage (n=31), > 15% anterior insula damage (n=9), healthy controls (n=33)	Self-report measures (Toronto Alexithymia Scale – TAS-20)	Anterior insula damage predicted increased alexithymia after controlling for anterior cingulate cortex damage and total lesion volume.
(Lang, 1994)	Review paper	Review paper	Discusses the role of cognition in interpreting nuanced emotional states, communicating them, making appropriate attributions, and responding accordingly.
(Laricchiuta et al., 2014)	Participants with low, borderline, or high alexithymia (n=60)	Self-report measures (Toronto Alexithymia Scale – TAS-20), Voxel-based morphometry (VBM; MRI) and Diffusion Tensor Imaging (DTI)	Lower right amygdala, left insula, and left parahippocampal gyrus and greater bilateral Crus 1 volumes corresponded to higher alexithymia scores.

(Mantani, Okamoto, Shirao, Okada, & Yamawaki, 2005)	Participants with high (n=10) and low (n=10) alexithymia.	Self-report measures (Toronto Alexithymia Scale – TAS-20) fMRI (Emotional Imagery task)	High relative to low alexithymia exhibited lower imagery vividness and emotional intensity during imagery for past and future sad events. High alexithymia group showed less posterior cingulate activity during past and future happy imagery, compared to rest and neutral imagery conditions.
(Okita et al., 2016)	Abstinent methamphetamine-dependent (n=23), healthy controls (n=17)	Self-report measures (Toronto Alexithymia Scale – TAS-20), PET measuring D2-receptor availability	Higher alexithymia scores in methamphetamine-dependent individuals. Significant positive relationship between D-2 receptor availability in ACC and anterior insula, and alexithymia, in controls, but non-significant negative relationship between D-2 receptor availability and alexithymia in methamphetamine-dependent group.
(Zaki, Davis, & Ochsner, 2012)	Healthy participants (n=16)	fMRI (Task 1: viewing emotional videos and rating emotion and eye gaze; Task 2: rating heartbeat, tones, and heartbeat with tones)	Activity in the anterior insula and inferior frontal operculum correlated with heartbeat monitoring (interoception), and scaled with trial by trial intensity of participants' emotional experience.
Autism spectrum disorder			
(Baron-Cohen & Wheelwright, 2004)	Adults with ASD (n=90), general population adults (n=90)	Self-report measures (Empathy Quotient – EQ)	ASD group scored significantly lower on EQ than controls.
(Berthoz & Hill, 2005)	Adults with ASD (n=27), normal	Self-report-measures (Toronto Alexithymia Scale – TAS-20,	Self-report on the BVAQ-B indicated that ASD was associated with cognitive, but not

	adults (n=35)	Bermond-Vorst Alexithymia Questionnaire – BVAQ-B, Beck Depression Inventory – BDI)	affective, alexithymia.
(Bird et al., 2010)	ASD (n=18); controls (n=18), matched on alexithymia scores	Self-report measures (Toronto Alexithymia Scale – TAS-20, Bermond-Vorst Alexithymia Questionnaire – BVAQ, Interpersonal Reactivity Index – IRI), fMRI (electric shock pain stimulation empathy task)	Both TAS-20 and BVAQ positively correlated with autism spectrum symptom severity.<br><br>Regarding the empathy for pain task, both groups had increased anterior insula activation, and the strength of this association was predictive of alexithymia.<br><br>No difference between ASD and controls in the degree of empathy related insula activation after controlling for alexithymia.
(Deschamps, Been, & Matthys, 2014)	Children with ASD (n=22), typically developing children (n=29)	Self-report measures (parent-completed SRS scores, GEM), computer task (Interpersonal Response Task), other tasks (Story Task)	On the GEM, both parents and teachers attributed lower cognitive empathy, but not affective empathy, to ASD children relative to controls.
(Hill, Berthoz, & Frith, 2004)	Adults ASD (n=27), biological relatives (n=49), adult controls (n=35)	Self-report measures (Toronto Alexithymia Scale – TAS-20, Beck Depression Inventory – BDI)	~50% of ASD adults exhibited Severe Impairment on the TAS-20, with 0% of controls exhibiting Severe Impairment.
(Silani et al., 2008)	ASD (n=15), controls (n=15)	Self-report measures (Toronto Alexithymia Scale – TAS-20, Bermond-Vorst Alexithymia Questionnaire – BVAQ, Interpersonal Reactivity Index – IRI); fMRI (view emotional or neutral pictures, and rate the internal	On the BVAQ-20, ASD differed from controls only on the poor insight and impaired cognition (i.e., cognitive alexithymia) subscales.<br><br>For both groups, while introspecting on internal emotional states, a positive

		emotion evoked)	correlation between parameter estimates in bilateral anterior insula and empathy, and a negative correlation with alexithymia, were observed. At a lower statistical threshold, ASD adults demonstrated hypoactivation of the anterior insula while rating how pleasant or unpleasant they felt after viewing negative affective images.
(Sucksmith, Allison, Baron-Cohen, Chakrabarti, & Hoekstra, 2013)	Adults ASD (n=314). parents of a child with ASD (n=297), controls (n=187)	Self-report measures (Empathy Quotient - EQ), behavioral task (Karolinska Directed Emotional Faces Task)	EQ scores were lower in ASD versus controls. On the Karolinska Directed Emotional Faces Task, ASD participants were less accurate at identifying a variety of emotional expressions relative to controls.
Psychopathy			
(Decety, Chen, Harenski, & Kiehl, 2013)	Incarcerated males classified as high (n=37), intermediate (n=44), and low (n=40) psychopathic traits, based on PCL-R self-report	Self-report measures (Psychopathy Checklist-Revised – PCL-R), fMRI (affective perspective taking task)	Intact insula responses when imagining self in pain, but reduced insula and amygdala activity and effective connectivity during "imagine-other" condition. High psychopathy group showed inverse relationship between PCL-R Factor 1 and insula activity when imagining other in pain.

(Decety, Chen, Harenski, & Kiehl, 2015)	Incarcerated male offenders divided into high (n=38), intermediate (n=67), and low/controls (n=50) psychopathic traits based on PCL-R self-report	Self-report measures (Psychopathy Checklist-Revised – PCL-R), fMRI (view clips of social dyad depicting interpersonal harm or assistance)	High psychopathy individuals more accurate than controls in identifying the emotion of social recipient during both helpful and harmful interactions and less accurate identifying emotion of agent. Decreased anterior insula activation in high psychopathy group when identifying the emotions of the agent.
(Grieve & Mahar, 2010)	Study 1, undergraduate students (n=75); Study 2, undergraduate students (n=275)	Self-report measures (Toronto Alexithymia Scale – TAS-20, Levenson Self-Report Psychopathy Scale – LSRP)	Bivariate correlations suggest that alexithymia is relatively less associated with primary versus secondary psychopathy.
(Hare, 1980)	Male prison inmates (n=143)	This is a measurement development study (Checklist for Psychopathy)	Factor 1 in this measure (which includes empathy as a core feature) accounts for the highest variance of all factors.
(Kroner & Forth, 1995)	Incarcerated male offenders (n=508)	Self-report measures (Toronto Alexithymia Scale – TAS-20, Basic Personality Inventory – BPI, Psychopathy Checklist – PCL-R)	Total PCL-R total score was negatively correlated with TAS-20 total score, as well as negatively correlated with the Emotional Understanding factor of the TAS-20.
(Jonason & Krause, 2013)	Online participants (n=322)	Self-report measures (Dark Triad Traits, Toronto Alexithymia Scale – TAS-20, Basic Empathy Scale)	Psychopathy-related traits correlated with limited overall empathy, difficulty describing feelings, and less externally oriented thinking. Different facets of alexithymia predicted different forms of limited empathy and psychopathy related traits.

(Lander & Lutz-Zois, 2012)	College students (n=104)	Self-report measures (Toronto Alexithymia Scale – TAS-20, Levenson Self-Report Psychopathy Scale – LSRP, Psychopathic Personality Inventory-Revised – PPI-R, State-Trait Anxiety Inventory – STAI)	Primary psychopathy positively correlated with the TAS-20 total score, as well as with all TAS-20 subscales. Secondary psychopathy was only correlated with the TAS-20 externally oriented thinking subscale.
(Louth, Hare, & Linden, 1998)	Female offenders (n=37)	Self-report measures (Psychopathy Checklist-Revised – PCL-R, Toronto Alexithymia Scale – TAS-26)	Positive correlations were found between PCL-R Factor 2 (i.e., social deviance) and TAS total scores, as well as between the PCL-R Factor 2 and TAS Factor 1 (i.e., inability to distinguish and describe feelings).
(Marsh et al., 2013)	Adolescents with oppositional defiant disorder or conduct disorder and high psychopathic traits (n=14), healthy controls (n=21)	Self-report measures (Psychopathy Checklist-Youth – PCL:YV), fMRI (pain perspective taking task)	Reduced insula response in patient group compared to control group when imaging others in pain.
(Meffert, Gazzola, Den Boer, Bartels, & Keysers, 2013)	Psychopathic offenders (n=18), controls (n=26)	Self-report measures (Psychopathy Checklist-Revised – PCL-R, Psychopathic Personality Inventory, fMRI (view clips of emotional interactions between two hands)	Decreased insula response during observation of emotionally relevant interactions in psychopathic offenders.
(Seara-Cardoso, Viding, Lickley, & Sebastian, 2015)	Males with no reported psychiatric illness (n=46)	Self-report measures (Self-Report Psychopathy Scale, Short Form – SRP-SF), fMRI (empathy for pain task)	Affective-interpersonal psychopathy traits were negatively associated with insula response while empathizing, where as lifestyle-antisocial traits were positively associated.

(Winter, Spengler, Bermpohl, Singer, & Kanske, 2017)	Violent male offenders (n=29),<br><br>controls (n=32)	Self-report measures (Toronto Alexithymia Questionnaire – TAS-20, Buss-Perry-Aggression-Questionnaire –BPAQ, Reactive-Proactive Aggression Questionnaire –RPQ), Social Video Task (includes questions on empathy, compassion, theory of mind reasoning, and factual reasoning)	Violent offenders showed lower empathy responses to emotional videos, which correlated with self-reported aggression (BPAQ & RPQ). This effect was mediated by alexithymia (TAS-20).
Narcissistic personality disorder			
(Baskin-Sommers, Krusemark, & Ronningstam, 2014)	Review paper	Review paper	Reviews literature on both emotional and cognitive empathy in the context of NPD. Literature suggests that for NPD individuals, emotional empathy deficits are more pronounced relative to cognitive empathy deficits.
(Dimaggio et al., 2007)	Narcissistic personality disorder (n=2), avoidant personality disorder (n=2)	Clinician-rated assessment	NPD patients (and one APD patient) demonstrated difficulties identifying emotions.
(Fan et al., 2011)	Non-clinical subjects exhibiting high (n=11) and low (n=11) narcissism	Self-report measures (Symptom Checklist-90-Revised – SCL-90-R), Narcissism Inventory – NI, Toronto Alexithymia Scale –TAS-20)	High narcissism group demonstrated higher scores on TAS-20 and SCL-90-R, and had decreased positive signal change from non-empathy to empathy in the right anterior insula.
(Jonason & Krause, 2013)	Online survey volunteers (n=322)	Self-report measures (Dark Triad Dirty Dozen – DTDD, Toronto Alexithymia Scale – TAS-20, Basic	Narcissism uniquely predicted the difficulty identifying feelings TAS-20

		Empathy Scale)	subscale.
(Marissen, Deen, & Franken, 2012)	NPD patients (n=20), patients with a Cluster C spectrum personality disorder (n=20), healthy controls (n=20)	Self-report measures (Interpersonal Reactivity Index – IRI, behavioral task (facial emotion recognition)	No empathy deficits found on the IRI. NPD patients performed worse in facial emotion recognition compared to other groups. NPD patients also showed a specific deficit in fear and disgust emotions.
(Ritter et al., 2011)	NPD patients (n=47), controls (n=53)	Self-report measures (Interpersonal Reactivity Index – IRI), behavioral task (Multifaceted Empathy Test – MET, Movie for the Assessment of Social Cognition – MASC)	NPD was associated with emotional empathy impairments on the MET. On the MASC, NPD patients did not exhibit cognitive empathy deficits relative to BPD patients and controls.
(Schimmenti et al., 2017)	Adults (n=777)	Self-report measures (Dark Triad Dirty Dozen – DTDD, Toronto Alexithymia Scale – TAS-20, Empathy Quotient – EQ), behavioral task (Reading the Mind in the Eyes Test-Revised version)	The DTDD traits were positively associated with alexithymia and negatively associated with empathy. Difficulty identifying feelings and reduced emotional reactivity were significant predictors of narcissism and the DTDD total score.
(Schulze et al., 2013)	NPD patients (n=17), controls (n=17)	Self-report measures (Interpersonal Reactivity Index – IRI), MRI (measure gray matter volume)	NPD patients had smaller GM volume in the left anterior insula relative to controls. Independent of group, GM volume in the left anterior insula was positively associated with emotional empathy
Borderline personality disorder			

(Beeney, Hallquist, Ellison, & Levy, 2016)	Females with BPD (n=17), controls (n=21)	Self-report measures (Differentiation of Self Inventory – DSI), behavioral task (Self-Aspects Card Sort Task), fMRI (evaluate personality traits of the self and others)	Insula activation mediated poorer consistency in rating the self and others in BPD.
(Bungert et al., 2015)	Females with BPD (n=20), controls (n=20)	Pain task (high temperature pain administration), fMRI (cyberball paradigm)	Social exclusion led to higher reported physical pain sensitivity in both groups, and this was accompanied by enhanced anterior insula activation. In BPD, physical pain processing after exclusion was linked to enhanced posterior insula activation.
(Dziobek et al., 2011)	Sample 1: BPD (n=21), controls (n=21), sample 2: BPD (n=30), controls (n=29)	Self-report measures (Interpersonal Reactivity Index – IRI), task (Multi-Faceted Empathy Test), fMRI, skin conductance response	No empathy impairments were found in BPD on self-report measures. BPD associated with cognitive and emotional empathy deficits on Multi-Faceted Empathy Test. BDP associated with reduced activity in right middle insular cortex during emotional empathy.
(Flasbeck, Enzi, & Brüne, 2017)	Females with BPD (n=50), controls (n=48)	Self-report measures (Toronto Alexithymia Scale –TAS-20, Interpersonal Reactivity Index – IRI, Childhood Trauma Questionnaire – CTQ), behavioral task (empathy task for which participants rated level of physical and psychological pain from both first- and third-person perspectives)	BPD rated psychological pain as more intense in the first-person versus third-person perspective. Controls did not differentiate between perspectives. The impact of early adversity on physiological pain empathy was mediated by alexithymia.

(Frick et al., 2012)	Females with BPD (n=21), female controls (n=20)	Self-report measures (Affective Lability Scale, Beck Depression Inventory), fMRI (Reading the Mind in the Eyes task)	BPD performed better than controls on positive and negative affect emotion recognition. BPD evidenced decreased activation in the insula during affective eye gazed relative to controls.
(Glenn & Klonsky, 2009)	Sample 1: undergraduate students (n=273), sample 2: undergraduate students (n = 30)	Self-report measures (DERS, DASS-21, PANAS, MSI-BPD)	Emotion dysregulation accounts for unique variance with BPD.
(Kalpakci, Vanwoerden, Elhai, & Sharp, 2016)	Adolescent inpatients BPD (n=107), controls (n=145)	Semistructured interview (CI-BPD), computerized diagnostic measure (C-DISC), self-report measures (BES, DERS), parent-report measure (CBCL), task (MASC)	BPD was associated with higher affective empathy than controls. Increased emotional dysregulation was associated with decreased affective empathy in BPD.
(Koenigsberg et al., 2009)	BPD (n=19), controls (n=17)	Task (International Affective Pictures System, pictures with social-emotional content)	When viewing negative pictures versus rest, BPD patients evidenced less difference in activation in the insula relative to controls.
(Ridings & Lutz-Zois 2014)	Undergraduate students (n=100)	Self-report measures (Levenson Self-Report Psychopathy Scale – LSRP, Toronto Alexithymia Scale – TAS-20, Coolidge Axis II Inventory – CATI, Difficulties in Emotion Regulation Scale – DERS)	Alexithymia positively correlated with both emotional dysregulation and BPD tendencies.

(Wolff, Stiglmayr, Bretz, Lammers, & Auckenthaler, 2007)	Females with BPD (n=30), female controls (n=28)	The MINITOR program: handheld device that administers self-report items while participants engage in everyday life	BPD patients exhibited more intense unpleasant emotions, greater difficulties with identifying their emotions, and increased aversive inner tension, relative to controls. Increased aversive inner tension was associated with increased difficulty identifying internal emotions.
